# Cross-Section Calculations for Electron-Impact Ionization of Pyrimidine Molecule and Its Halogenated Derivatives: 2-Chloropyrimidine, 5-Chloropyrimidine, 2-Bromopyrimidine and 5-Bromopyrimidine

**DOI:** 10.3390/molecules30010006

**Published:** 2024-12-24

**Authors:** Bożena Żywicka, Paweł Możejko

**Affiliations:** Faculty of Applied Physics and Mathematics, Gdańsk University of Technology, ul. Gabriela Narutowicza 11/12, 80-233 Gdańsk, Poland; bozmozej@o365.pg.edu.pl

**Keywords:** electron-impact ionization, binary-encounter-Bethe model, pyrimidine, 2-chloropyrimidine, 5-chloropyrimidine, 2-bromopyrimidine, 5-bromopyrimidine, electron scattering

## Abstract

The total cross-sections for the single electron-impact ionization of pyrimidine (C_4_H_4_N_2_), 2-chloropyrimidine (2-C_4_H_3_ClN_2_), 5-chloropyrimidine (5-C_4_H_3_ClN_2_), 2-bromopyrimidine (2-C_4_H_3_BrN_2_) and 5-bromopyrimidine (5-C_4_H_3_BrN_2_) molecules have been calculated with the binary-encounter-Bethe model from the ionization threshold up to 5 keV. The input data for the BEB calculations concerning electronic structure of the studied targets have been obtained with quantum chemical methods including the Hartree–Fock (H-F) and the outer valence Green function (OVGF) methods. The calculated cross-section for the ionization of the pyrimidine molecules due to electron impact is compared with available experimental and theoretical data. The question of the magnitude the pyrimidine ionization cross-section is also discussed, as is the efficiency of the ionization process of studied halogenated derivatives of pyrimidine.

## 1. Introduction

A detailed and deep understanding of electron scattering from atoms and molecules is crucial for the interpretation the relevant phenomena occurring in many different environments, including natural and technological ones [1,2]. Among all possible processes accompanying the interaction of electrons with molecules, electron-impact ionization and dissociative electron attachment (DEA), leading to the formation of positive and negative ions, respectively, seem to be very important due to the role these products play in chemical reactions. Also, in the context of the interaction of primary ionizing radiation (α, $\beta^{\pm }$, γ and etc.) with biomatter, secondary products such as low energy electrons, ions and radicals play an important role in the final effect of irradiation. The discovery of the fact that low-energy (0–20 eV) secondary electrons can induce DNA damage, including single- and double-strand breaks by the dissociative electron attachment (DEA) process, indicates the need to take their contribution into account in radiotherapy [3,4,5,6]. Such secondary low-energy (0–100 eV) electrons are created in large quantities by ionizing radiation (∼4 × $10^{4}$ per 1 MeV of primary ionizing radiation) [7]. In the next stages of radiolysis, they may lead to further ionization of the medium or its capture in the electron attachment process. The effect of the interaction of primary ionizing radiation and secondary products with living cells and the DNA they contain can be modeled in Monte Carlo simulations. For example, the Geant4-DNA package [8,9] allows its inclusion into simulations of many electron interaction processes with biological environments, like elastic electron scatterring (7.4 eV–1 MeV), electron-impact electronic excitation (9 eV–1 MeV), electron-impact vibrational excitation (2–100 eV), electron-impact ionization (11 eV–1 MeV) and electron attachment by studied target (4–13 eV). This Monte Carlo toolkit enables thorough investigations covering the physical, pre-chemical and chemical phases of early DNA damage induced by primary ionizing radiation, including indirect DNA damage induced by secondary ionizing particles, including low-energy electrons [10]. For such simulations, a complete set of high-quality data on the efficiency of the interaction, most often described by the cross-section for a given reaction, of primary ionizing radiation and secondary products is necessary. Since it is not possible to determine such data for all relevant chemical compounds and all possible interaction energies and all scattering channels in a reasonable time by means of measurements or advanced quantum-mechanical numerical calculations, reliable simple models are necessary. In order to develop such models, it is necessary to conduct thorough experimental and theoretical studies concerning, among others, the interactions of electrons with biomatter and simple molecular analogues of its components (see e.g., Refs. [11,12] and references therein).

**Figure 1:**
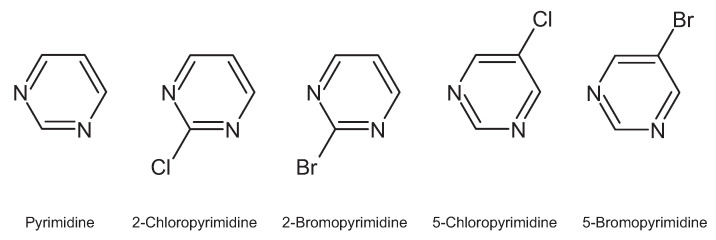
Schematic drawing of pyrimidine molecules and its halogenated derivatives.

Pyrimidine (1,3-diazine) is a six-membered, aromatic, heterocyclic organic compound in which two CH groups of the benzene molecule are replaced by nitrogen atoms at positions (1) and (3) of the ring (Figure 1). In studies on the damage to biological tissues by ionizing radiation in cancer radiotherapy, pyrimidine has been widely recognized as a precursor and/or analog of the nucleobases cytosine, thymine and uracil, which are the structural units of DNA and RNA. Moreover, derivatives of halogenated pyrimidines can incorporate into DNA and act as radiosensitizers making it more sensitive to radiation damage. Therefore, intensive research is being carried out on new compounds based on pyrimidine and its derivatives, which can act as radiosensitizers, potential antioxidants and selective anticancer agents [13,14,15]. Taking all the above facts into account, it is not surprising that relatively many studies have been carried out on electron scattering on pyrimidine molecules, including electron-impact ionization studies.

The theoretical and experimental work to date on collisional interactions of electrons with pyrimidine molecules can be briefly summarized as follows. Total electron-scattering cross-section of pyrimidine was measured using the linear transmission spectrometer for energies from 20 eV to 5 keV [16] and for electron energies ranging from 8 eV to 500 eV using a magnetically confined experimental setup [17]. The same work reported integral elastic and inelastic electron-scattering cross-sections calculated with the spherical complex optical potential model [16]. The stopping power for electron in pyrimidine was calculated based on measured the mean energy loss and theoretical total and elastic cross-sections obtained with the screening-corrected additivity rule method [18]. Relatively many works concern measurements of the differential cross-section for elastic electron scattering from pyrimidine [19,20,21,22,23]. Differential cross-sections for elastic electron scattering on pyrimidine for selected electron energies ranging from 1 eV to 20 eV and at scattering angles up to 180° have also been measured using an electron-impact spectrometer with hemispherical analyzer and a magnetic angle changer [24]. Experimental integral elastic cross-section as a function of electron energy in the range 0.1–14 eV, as well as calculated ones with the-fixed-nuclei Schwinger variational and R-matrix methods have been also reported [24]. Experimental, as well as theoretical works also concerned electronic and vibrational excitation of pyrimidine by electron impact [25,26,27]. Total and elastic cross-sections for electron scattering from pyrimidine molecule were also calculated with a spherical complex optical potential formalism for an extensive energy range of 10 eV to 5 keV [28]. Resonances occurring due to low energy electron interaction with pyrimidine in the elastic channel were studied with R-matrix method [29]. Cross-sectionsdata set, including among others total, differential and total elastic cross-sections, for an impact of electron in the energy range between 10 eV and 1 keV on pyrimidine molecules has been reported [30]. Very recently, a comprehensive set of calculated scattering cross-section data of pyrimidine, including elastic one computed with the single-centre-expansion formalism, for impinging electron energies ranging from 1 eV to 5 keV has been also reported [31].

Of course, a relatively large number of works concern the ionization of the pyrimidine molecule induced by collisions with electrons. The experimental total and partial cross-sections for electron-impact ionization of pyrimidine molecule were obtained employing the total ion collection technique and the quadrupole mass spectrometer from the ionization threshold up to 150 eV [32]. Total ionization cross-section of pyrimidine was also determined from measured doubly differential electron-impact ionization cross-section of pyrimidine [33]. Absolute total and partial dissociative ionization cross-sections were also measured for electron impact energies ranging from 70 to 400 eV through time of flight mass spectrometry and calculated using semi-empirical approach [34]. Total ionization cross-section obtained by integrating the measured double-differential ionization cross-sections was also reported [30]. Theoretical studies on the calculations of electron-impact ionization cross-section of pyrimidine molecules include computations using the spherical complex optical potential formalism with complex scattering potential ionization contribution method [35] and quantum-mechanical study based on the first Born approximation [36]. Total and/or partial ionization cross-sections due to electron impact calculated with the binary-encounter-Bethe method have been also reported in several works [30,31]. Please note that preliminary version of our calculations of electron-impact ionization cross-section of pyrimidine molecules has been also presented previously [37].

Much less work has been conducted on the interaction of electrons with halogenated pyrimidines. Here we note studies on the formation of temporary anion states due to low energy impact on haloprymidines [38] and the theoretical study of shape resonance states occurring during the interaction of low-energy electrons with halopyrimidines [39].

The objective of the present work is to provide carefully and precisely calculated electron-impact ionization cross-sections for pyrimidine and its simple halogenated derivatives i.e., 2-chloropyrimidine, 5-chloropyrimidine, 2-bromopyrimidine and 5-bromopyrimidine.

## 2. Results and Discussion

**Figure 2:**
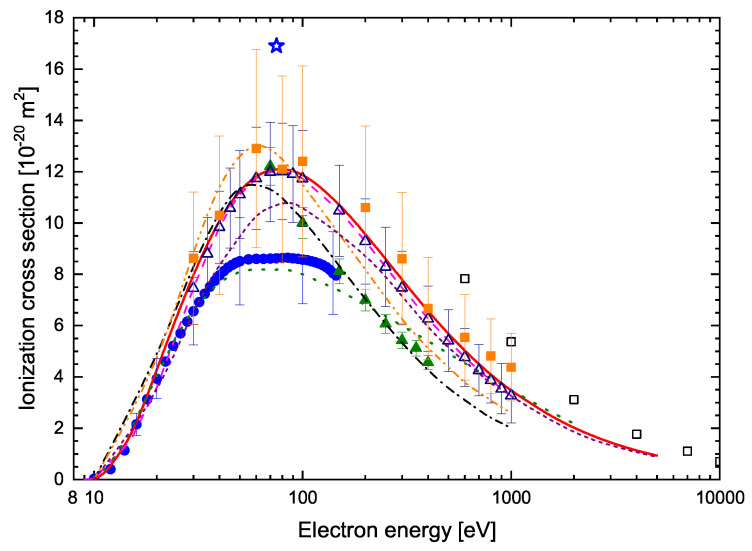
Comparison of the ionization cross-sections due to electron impact on pyrimidine molecule. Experimental ICS: full circles [32]; full triangles [34]; open triangles [30]; full squares [33]. Theoretical: solid line, present BEB results; dash line [30]; dot line [35]; dash dot line [36]; dash dot dot line [34]; short dash line [31]. For a comparison, experimental cross-sections for the electron-impact ionization of a benzene molecule are also shown: open star [40]; open squares [41].

The numerical values of the calculated cross-sections for the electron-impact ionization of studied targets, for energies ranging from the ionization threshold to 5 keV, are summarized in Table 1. The ionization cross-section for pyrimidine molecules computed in this work is shown in Figure 2. This is compared with the available experimental results [30,32,33,34]. The results of the present calculations are in very good agreement with the experimental results obtained by integrating the measured double-differential ionization cross-sections [30]. Fairly good agreement is also seen between the present results and the recently evaluated total ionization cross-section, also obtained from double-differential electron impact ionization cross-sections [33]. The latter are slightly higher, but taking into account the declared measurement uncertainty of 30%, these differences can be considered as approximately insignificant. However, the present data are higher by about 20% than the results measured through time of flight mass spectrometry [34]. This does not apply only for the energies from the ionization threshold up to 70 eV, at which the results of both the mentioned experiments [30,34] and our calculations are in almost perfect agreement. This may indicate that in the experiment with the time of flight mass spectrometer [34] at higher impact-electron energies, not all formed ions were recorded and the contribution from the inner-shell ionization was also slightly underestimated. The results obtained with the use of the total ion collection technique [32], except the low energy range from the ionization threshold up to 30 eV, are much smaller than the predictions of the present calculations and other experimental data [30,33,34]. This difference is even up to 50%.

In the same figure (Figure 2), for comparison, the total cross-sections for the ionization of pyrimidine determined in all previous theoretical calculations [30,31,34,35,36] are shown. All calculated pyrimidine ionization cross-sections have very similar shapes. However, there are visible differences in the maximum positions and predicted cross-section values. In particular, the results of semi-empirical calculations [34] and those based on the first Born approximation [36] indicate a maximum position at about 60 eV, while the others predict maxima at about 80 eV. The present results are in good agreement with the previously calculated BEB ionization cross-section [30]. The minor discrepancies are due to small differences in the calculations of the electronic structure of pyrimidine in both studies. However, the results of the latest calculations [31], also using the BEB method, are systematically lower than our data and the earlier results of BEB method calculations [30]. The maximum differences reach up to 10%. It is worth adding that these results [31] were obtained using binding energies calculated at the HF level with a cc-pVTZ basis set [31] without taking into account any correlation energy, which usually leads to an underestimation of the cross-section value in the BEB method. The results of semi-empirical calculations [34] and those obtained using the first Born approximation [36] are in satisfactory agreement with the results of the remaining calculations only for the energies in the range from the ionization threshold to about 30 eV. They predict, as we mentioned above, the location of the ionization maximum at an energy of about 60 eV. Moreover, the semi-empirical calculations [34] gave a maximum higher than the present ones by about 10%, while the results obtained in the first Born approximation [36] for energies greater than 80 eV are systematically lower than the present ones by about 10%. The results obtained with the spherical complex optical potential formalism and complex scattering potential ionization contribution method [35] are the least consistent with the other calculations. However, they are in good agreement with the ionization cross-section measured with the use of the total ion collection technique [32].

Since, as can be seen in Figure 2, there are quite significant discrepancies between the predictions of theoretical calculations and the results of various experiments, an important question is which of these results is the most realistic. To provide a preliminary answer to this question, the experimentally determined ionization cross-sections of benzene molecules [40,41] are depicted in Figure 2. Benzene is isoelectronic with pyrimidine and therefore such a comparison may be useful in this analysis to some extent. Of course, the same number of electrons in a molecule does not mean the same total cross-section for ionization. The most important things here are the molecular electronic structure and, consequently, the binding energies of the individual electrons. Taking into account the differences in the structure of benzene and pyrimidine, it can be expected that the ionization cross-section of benzene will be higher than that of pyrimidine. Despite this, it can be expected that the ionization results of benzene and pyrimidine should not differ drastically. Hence, analyzing the data presented in Figure 2, it seems that experimental results measured with the total ion collection technique [32], which differ from those for benzene by almost twice the amount of ionization, can be at least slightly underestimated.

**Figure 3:**
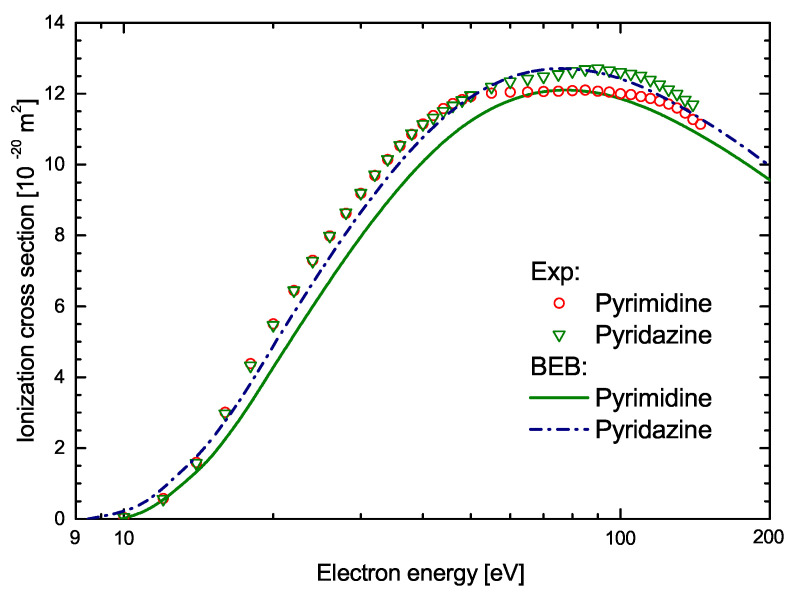
Comparison of the electron-impact ionization cross-sections for pyridazine and pyrimidine molecules. Experimental and theoretical data for pyridazine are from Ref. [42]. The solid line represents present theoretical data for pyrimidine and the open circles are experimental data [32] for this compound normalized using the calculated results.

Figure 3 presents computed ICS for pyrimidine molecules compared with previously reported theoretical BEB data for pyridazine [42]. Since both compounds are isomeric, it is not surprising that the ionization cross-sections calculated for them are very similar. The cross-section for pyridazine has slightly higher values in the whole range of compared energies. Additionally, in Figure 3, experimental ICSs for pyridazine measured with quadrupole mass spectrometer are shown [42]. It is important to stress here that this data are normalized. Specifically, the total measured cation efficiency was normalized to the calculated cross-section, assuming that the measured cross-section should be equal at its maximum to the calculated maximum. For comparison, the experimental ionization cross-section for pyrimidine [32] normalized to the present computed ICSs are also depicted in Figure 3. Normalization was performed assuming that the maximum in the measured cross-section occurring at 85 eV should be equal to the maximum calculated value, i.e., $12.10\times 10^{-20}$ m^2^. As can be seen in Figure 3, the resulting normalized experimental cross-section is in quite good agreement with our calculation results for all other common energies.

**Figure 4:**
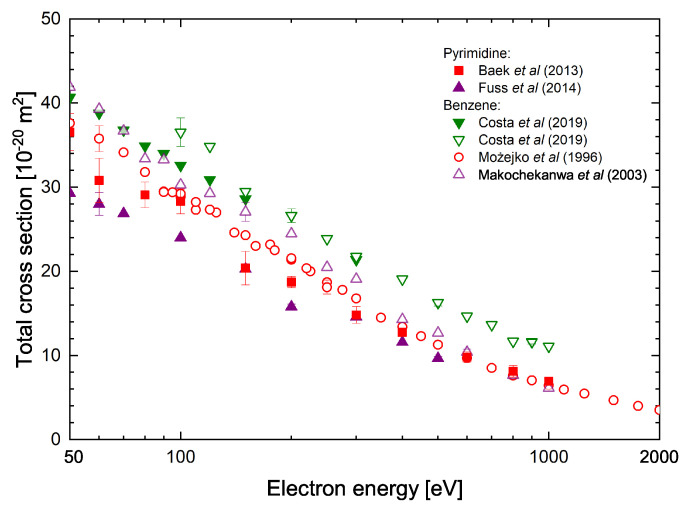
Comparison of the total scattering cross-sections (TCS) for electron scattering from benzene (C_6_H_6_) and pyrimidine (C_4_H_4_N_2_) molecules measured for intermediate energy range. TCS for pyrimidine molecules: full squares [16]; full triangles [17]. TCS for benzene molecules: open circles [43]; open triangles [44]; open and full down triangles [45].

To determine whether the ionization cross-section of pyrimidine should have values similar to those of pyridazine and not differ too much from the cross-sections of benzene in Figure 4, we compared the absolute total cross-sections for electron scattering from pyrimidine [16,17] and benzene molecules [43,44,45]. Total cross-section (TCS) is a quantity giving valuable information on the overall scattering phenomena. Moreover, TCS can be usually measured in absolute scale over wide energy range without any normalization procedure and can thus serve as quantitative tests of the reliability of theoretical models.

Of course, the TCS includes the contribution from all scattering channels opened at a given collision energy. However, in our analysis, it is important to note that at higher collision energies (above ∼100 eV), the dominant contribution to the scattering process comes from elastic collisions and overall ionization processes. As can be seen from Figure 4, the experimental TCS data for benzene are in reasonable agreement. The differences between these results do not exceed 20%. TCSs for pyrimidine were measured with two different electron-transmission experiments in which results were obtained using the electrostatic apparatus with highly reduced magnetic field [16], and with a system using magnetically confined electron beam [17]. Above 60 eV, both TCS data sets [16,17] are very similar, and importantly, they do not differ significantly from the results for benzene, especially from older experimental data [43,44]. In the latter case, the differences between these results are generally within the total declared measurement uncertainties. It is worth to note that experimental electron-impact differential scattering cross-sections for elastic scattering from benzene and pyrimidine are quite similar [46]. This of course is not a direct proof, but taking into account the previously described comparative analysis of the results for pyrimidine ionization, it can be expected that the presented ICS results calculated for pyrimidine molecules are very likely to have reliable values. However, further studies of the efficiency of the ionization processes in electron collisions with pyrimidine, primarily experimental, are necessary to finally resolve this problem.

**Figure 5:**
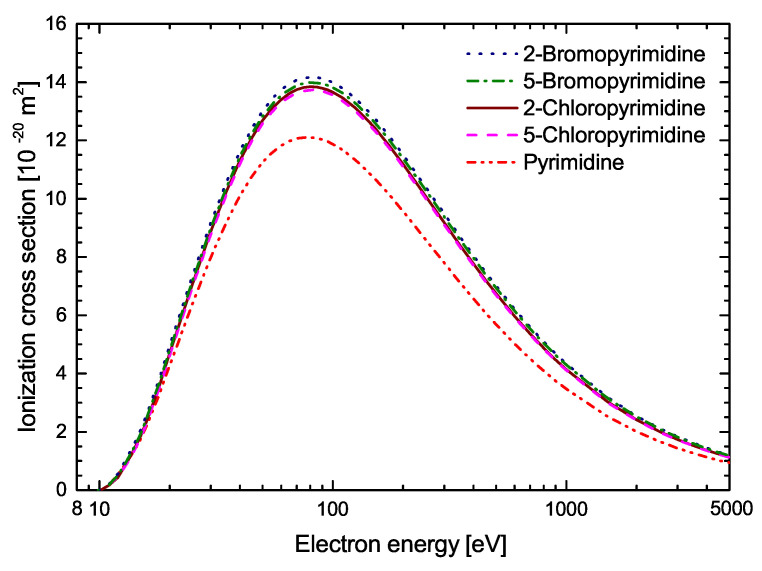
Comparison of the calculated with BEB method ionization cross-sections induced by electron collisions with pyrimidine (dash dot dot line), 2-chloropyrimidine (solid line), 5-chloropyrimidine (dash line), 2-bromopyrimidine (dot line) and 5-bromopyrimidine (dash dot dot line) molecules.

In Figure 5, calculated in this work, within BEB approximation, the total cross-sections for electron-impact ionization of pyrimidine, 2-chloropyrimidine, 5-chloropyrimidine, 2-bromopyrimidine and 5-bromopyrimidine are presented and compared. For all the studied compounds, a maximum of the ICS is located at around 80 eV. For the halogenated derivatives of pyrimidine, the cross–section values are very similar in the entire energy range studied. However, the highest cross-section is for 2-bromopyrimidine, reaching a maximum of $14.17\times 10^{-20}$ m^2^. For 5-bromopyrimidine, the maximum ICS value is $13.99\times 10^{-20}$ m^2^. For 2-chloropyrimidine and 5-chloropyrimidine, the maximum ICS value is similar and amounts to $13.84\times 10^{-20}$ m^2^ and $13.73\times 10^{-20}$ m^2^, respectively. This tendency in the dependencies between the cross-section values for these compounds is visible from almost the ionization threshold up to 500 eV. In the case of 2-chloropyrimidine and 5-chloropyrimidine, their respective ionization cross-sections almost coincide at incident electron energies above 500 eV. Obviously, a similar dependence can be observed for 2-bromopyrimidine and 5-bromopyrimidine. This reflects the fact that at higher energies the small differences in the electronic structure of these isomeric pairs do not play a significant role. However, they do affect the ionization efficiency at lower energies. The electron-impact ionization cross-section of pyrimidine is significantly lower than the ICSs for its halogenated derivatives and reaches a maximum of $12.10\times 10^{-20}$ m^2^. This indicates a potentially high efficiency of the response of halogenated pyrimidines and radiosensitizers based on them to ionizing radiation in comparison with pyrimidine.

## 3. Computational Methods

Ultimately, the theoretical method and numerical procedures employed in the present work are almost the same as in our previous studies, in which we have calculated electron-impact ionization cross-sections for building blocks of DNA and RNA [47,48] and some simple biologically relevant molecules [42,49,50]. For this reason, below we provide a rather concise and short description of the method and calculations used in this work.

Electron-impact ionization cross-section (ICS) has been calculated with the binary-encounter-Bethe (BEB) method [51,52]. The BEB model is the simplified version of the binary-encounter-dipole (BED) method [51] that combines the binary-encounter theory [53] suitable for low electron impact energies with the Bethe theory valid for high electron-impact energies [54]. While this method is still a model and semi-empirical approach to the ionization phenomenon, it does not contain any arbitrary parameters. Thus, the advantage of the BEB model is that all quantities necessary to calculate ICS have a well-defined physical meaning and can be calculated or evaluated quite accurately using standard quantum chemical methods. Of course, the accuracy of such quantum chemical calculations, and therefore the quality of the obtained ICS, depends to a large extent on the level of the theory applied and the basis sets used.

Within BEB formalism, the electron-impact ionization cross-section for a given molecular orbital can be calculated from the following formula:

(1) $$\sigma^{BEB}=\frac{S}{t+u+1}\left[\frac{\ln t}{2}\left(1-\frac{1}{t^{2}}\right)+1-\frac{1}{t}-\frac{\ln t}{t+1}\right],$$

where u=U/B, t=T/B, $S=4\pi a_{0}^{2}NR^{2}/B^{2}$, $a_{0}=0.5292$ Å, R=13.61 eV, *U* is the kinetic orbital energy, *T* is the energy of impinging electrons and *N* is the orbital occupation number. Finally, the total cross-section for electron-impact ionization, $\sigma_{ion}$, can be calculated as the sum of $\sigma^{BEB}$ for all molecular orbitals (MO):

(2) $$\sigma^{\mathrm{ion}}=\sum_{i=1}^{n_{MO}}\sigma_{n_{MO}}^{BEB}$$

To obtain the electron binding energies *B*, the kinetic energy of all molecular orbitals, *U*, and the orbital occupation numbers, *N*, quantum chemical computations have been performed using Gaussian code [55]. At first, we performed a geometry optimization of the studied molecules in the ground state within C_2*v*_ symmetry with the Hartree–Fock (HF) method with 6-311++G(2d,2p) Gaussian basis sets. In the next step, using the obtained geometries of the studied molecules, single point energy calculations were performed. The HF method with the same 6-311++G(2d,2p) Gaussian basis sets was also used in these calculations.

Since the energies of the highest occupied molecular orbitals (HOMO) obtained in this way usually are higher from those measured experimentally (even by about 1–3 eV), we also performed outer valence Green function calculations of correlated electron affinities and ionization potentials [56,57,58,59] using the GAUSSIAN code [55]. The resulting value of the ionization threshold for the pyrimidine molecule is 9.804 eV which is rather in good agreement with measured data: 9.9 eV [60], 9.71 eV [61], 9.35 eV [62], 9.73 eV [63] and 9.21 eV [64]. Please note that the data from Refs. [62,64] are photoelectron spectral values of the adiabatic ionization energy while the higher values are the vertical ionization energies [63]. For the remaining studied compounds that were calculated, the first ionization energies are as follows: 9.865 eV, 9.911 eV, 10.04 eV and 10.12 eV for 5-bromopyrimidine, 2-bromopyrimidine, 5-chloropyrimidine and 2-chloropyrimidine, respectively. The results of reported HF calculations for binding energies and orbital kinetic energies are presented in the Appendix A in the Table A1, Table A2 and Table A3. The values of the HOMOs energies calculated using the OVGF method are also listed there in the Table A4.

The BEB results obtained in the manner described are counting ionization cross-sections that include a contribution from a single ionization. Therefore, they should be considered as the lower limits to the experimental results in which the contributions from multiple ionizations can also be included [52]. It is worth to note here that, despite the previous remark, the BEB model is a well-known theoretical method that allows the obtainment of reliable ionization cross-sections for molecules. ICSs obtained with the BEB method are usually in good agreement (within ±15%) with experimental data [65,66,67].

## 4. Conclusions

The BEB method was used to calculate the ionization cross-sections for ionization induced by collisions with electrons of pyrimidine and its halogenated derivatives. All parameters necessary for the BEB method were carefully determined using quantum chemical methods. The cross-sections for ionization of pyrimidine are generally consistent with the results of experiments based on doubly differential electron-impact ionization cross-section measurements. The results obtained for pyrimidine are comparable with the results for its isomeric compound—pyridazine. The process of the ionization of halogenated derivatives of pyrimidine is much more efficient than the process of pyrimidine ionization. This indicates a possible potential use of these compounds as a base for new radiosensitizers. The need for further experimental and theoretical work on the ionization of pyrimidine and its derivatives by electron interaction was also pointed out.

## Figures and Tables

**Table 1:** Ionization cross-section for electron-impact ionization of pyrimidine and its halogenated derivatives in units of $10^{-20}$ m^2^.

Energy [eV]	C_4_H_4_N_2_	2-C_4_H_3_ClN_2_	5-C_4_H_3_ClN_2_	2-C_4_H_3_BrN_2_	5-C_4_H_3_BrN_2_
9.804	0.00000				
9.865					0.00000
9.900	0.00934				0.00355
9.911				0.00000	
10.00	0.01920			0.00883	0.01366
10.04			0.00000		
10.12		0.00000			
11	0.1877	0.1697	0.1930	0.2058	0.1899
12	0.5286	0.4370	0.4573	0.5588	0.5013
13	0.9283	0.8912	0.8583	1.052	0.9616
14	1.319	1.368	1.318	1.540	1.437
15	1.744	1.844	1.775	2.050	1.904
16	2.244	2.402	2.342	2.628	2.508
17	2.753	2.981	2.924	3.223	3.099
18	3.267	3.547	3.485	3.798	3.668
19	3.785	4.131	4.053	4.393	4.244
20	4.283	4.692	4.610	4.959	4.805
25	6.380	7.055	6.960	7.341	7.168
30	7.992	8.874	8.775	9.175	8.995
35	9.199	10.26	10.15	10.57	10.39
40	10.11	11.31	11.21	11.63	11.45
45	10.77	12.10	11.99	12.43	12.24
50	11.25	12.68	12.57	13.01	12.82
55	11.59	13.11	12.99	13.43	13.24
60	11.82	13.40	13.29	13.73	13.55
65	11.97	13.61	13.50	13.94	13.75
70	12.06	13.74	13.63	14.07	13.88
75	12.10	13.81	13.70	14.14	13.96
80	12.10	13.84	13.73	14.17	13.99
85	12.08	13.83	13.72	14.16	13.98
90	12.02	13.79	13.69	14.12	13.94
95	11.95	13.73	13.63	14.06	13.89
100	11.87	13.65	13.55	13.98	13.81
110	11.67	13.46	13.36	13.79	13.63
125	11.33	13.11	13.01	13.44	13.28
150	10.72	12.46	12.37	12.79	12.64
175	10.13	11.80	11.72	12.13	12.00
200	9.568	11.18	11.10	11.51	11.38
250	8.594	10.09	10.01	10.40	10.29
300	7.789	9.171	9.105	9.477	9.374
350	7.122	8.407	8.347	8.701	8.608
400	6.564	7.763	7.708	8.046	7.960
450	6.090	7.214	7.163	7.487	7.407
500	5.683	6.741	6.694	7.004	6.930
600	5.020	5.968	5.926	6.213	6.148
700	4.503	5.362	5.325	5.592	5.534
800	4.089	4.874	4.841	5.091	5.039
900	3.748	4.473	4.443	4.678	4.630
1000	3.463	4.137	4.109	4.331	4.287
1500	2.530	3.031	3.011	3.186	3.155
2000	2.009	2.411	2.395	2.542	2.516
2500	1.674	2.011	1.998	2.124	2.104
3000	1.440	1.731	1.720	1.831	1.813
3500	1.266	1.523	1.513	1.612	1.597
4000	1.131	1.361	1.353	1.443	1.429
4500	1.024	1.233	1.225	1.307	1.295
5000	0.9359	1.127	1.120	1.196	1.185

## Data Availability

The original contributions presented in this study are included in the article. Further inquiries can be directed to the corresponding author.

## Abbreviations

The following abbreviations are used in this manuscript:

BEB	Binary–encounter–Bethe
BED	Binary–encounter-dipole
HF	Hartree–Fock
ICS	Ionization cross-section
OVGF	Outer valence Green function
TCS	Total cross-section

## Appendix A. Binding Energies, B, Kinetic Orbital Energies U and HOMOs Energies of the Studied Targets

This appendix summarizes the data that were calculated in this work on the binding energies, *B*, and the kinetic orbital energies, *U*, of the compounds studied. All results are given in electronvolt. In Appendix A.1, the results obtained with the Hartree–Fock method with 6-311++G(2d,2p) Gaussian basis sets are tabulated in Table A1, Table A2 and Table A3. The ionization energies of the selected highest occupied molecular orbitals computed with the outer valence Green function method are tabulated in Table A4 of Appendix A.2. All calculations were performed with Gaussian code [55].

### Appendix A.1. Calculated with the HF Method Binding Energies B, Kinetic Orbital Energies U of the Studied Targets

**Table A1:** Binding energies *B* (eV) and kinetic orbital energies *U* (eV) calculated at the HF level for pyrimidine molecule.

Orbital No.	*B* (eV)	*U* (eV)
1	423.70	602.02
2	423.70	602.02
3	307.75	436.01
4	307.37	435.98
5	307.37	435.98
6	306.0	435.86
7	36.188	49.142
8	32.871	56.227
9	29.490	45.438
10	24.570	45.287
11	24.509	45.486
12	20.373	28.960
13	19.422	45.659
14	17.855	35.506
15	16.362	37.558
16	16.136	40.181
17	15.964	29.630
18	12.927	50.071
19	11.650	36.887
20	11.329	50.811
21	10.372	30.498

**Table A2:** Binding energies *B* (eV) and kinetic orbital energies *U* (eV) calculated at the HF level for 2-chloropyrimidine (column 2 and 3) and 5-chloropyrimidine molecules (column 4 and 5).

Orbital No.	*B* (eV)	*U* (eV)	*B* (eV)	*U* (eV)
1	2852.9	3731.1	2853.4	3731.1
2	424.16	602.00	424.13	602.02
3	424.16	602.01	424.13	602.02
4	309.71	436.13	308.08	436.01
5	307.84	435.99	307.95	435.87
6	307.84	435.99	307.82	435.97
7	306.44	435.85	307.81	436.08
8	287.58	593.54	288.09	593.55
9	218.69	560.89	219.20	560.91
10	218.61	561.98	219.12	562.03
11	218.60	562.07	219.12	562.05
12	37.053	49.944	36.686	49.013
13	33.520	56.392	33.318	56.271
14	31.419	61.358	32.204	62.570
15	29.034	58.689	28.678	60.286
16	25.241	45.425	25.224	45.271
17	24.247	51.509	24.414	51.024
18	20.215	34.408	20.679	30.373
19	20.116	45.191	20.211	45.843
20	17.870	41.651	17.228	54.826
21	16.944	31.629	16.981	37.236
22	16.822	37.613	16.661	30.324
23	16.212	50.782	16.589	41.560
24	13.492	54.721	14.032	51.316
25	13.221	50.513	13.350	53.848
26	12.666	60.695	13.279	61.505
27	12.154	37.355	12.082	36.944
28	11.733	54.286	11.625	52.252
29	10.351	39.768	10.204	41.309

**Table A3:** Binding energies *B* (eV) and kinetic orbital energies *U* (eV) calculated at the HF level for 2-bromopyrimidine (column 2 and 3) and 5-bromopyrimidine molecules (column 4 and 5).

Orbital No.	*B* (eV)	*U* (eV)	*B* (eV)	*U* (eV)
1	13334	16195	13335	16195
2	1772.9	3195.1	1773.5	3195.1
3	1592.2	3166.6	1592.8	3166.6
4	1592.2	3166.9	1592.7	3166.9
5	1592.2	3166.9	1592.7	3166.9
6	424.20	602.00	424.13	602.02
7	424.20	602.01	424.13	602.02
8	309.57	436.14	308.11	436.01
9	307.85	435.98	307.86	435.76
10	307.85	435.99	307.85	435.97
11	306.39	435.85	307.79	436.20
12	267.70	766.10	268.24	766.10
13	202.77	709.25	203.31	709.26
14	202.55	710.98	203.09	711.05
15	202.53	711.12	203.08	711.07
16	86.836	586.40	87.380	586.39
17	86.732	587.14	87.268	587.00
18	86.709	587.01	87.266	587.21
19	86.450	587.89	86.996	587.86
20	86.450	587.85	86.996	587.90
21	36.971	49.811	36.677	49.056
22	33.520	56.435	33.324	56.278
23	30.376	51.103	30.881	56.097
24	27.840	77.019	27.691	75.621
25	25.227	45.492	25.192	45.371
26	24.106	55.105	24.292	54.455
27	20.118	34.612	20.677	30.583
28	20.082	45.384	20.141	45.914
29	17.737	41.273	16.966	37.295
30	16.817	37.635	16.706	40.492
31	16.812	30.927	16.568	29.828
32	15.685	52.194	16.185	59.049
33	13.052	59.804	13.126	55.832
34	12.248	49.590	13.012	52.312
35	12.158	37.334	12.235	62.538
36	12.018	52.657	12.087	36.963
37	11.181	68.587	11.498	57.508
38	10.165	47.962	10.039	47.489

### Appendix A.2. Calculated with the OVGF Method HOMOs Energies of the Studied Targets

**Table A4:** Ionization energies (eV) of the HOMOs calculated with the OVGF method.

C_4_H_4_N_2_	2-C_4_H_3_ClN_2_	5-C_4_H_3_ClN_2_	2-C_4_H_3_BrN_2_	5-C_4_H_3_BrN_2_
	18.144	18.561	18.023	18.529
	17.851	17.923	17.768	17.808
18.302	16.220	15.839	16.047	15.062
17.293	15.278	15.129	15.107	15.080
16.204	15.027	15.045	14.982	15.006
14.693	14.678	14.890	14.100	14.767
14.630	11.845	13.108	11.655	12.290
14.565	12.382	12.297	11.603	11.591
11.331	11.675	11.690	11.599	11.583
11.327	11.640	11.632	10.497	11.204
10.461	10.178	10.075	10.301	10.184
9.804	10.120	10.040	9.911	9.865
